# Association between the gait pattern characteristics of older people and their two-step test scores

**DOI:** 10.1186/s12877-018-0784-5

**Published:** 2018-04-27

**Authors:** Yoshiyuki Kobayashi, Toru Ogata

**Affiliations:** 1 Digital Human Research Group, Human Informatics Research Institute, National Institute of Advanced Industrial Science and Technology, 2-3-26 Aomi, Koto-ku, Tokyo, 135-0064 Japan; 2 National Rehabilitation Center for Persons with Disabilities, 4-1 Namiki, Tokorozawa-shi, Saitama, Japan

**Keywords:** Principal component analysis, Mobility of older people, Two-step test, Gait pattern

## Abstract

**Background:**

The Two-Step test is one of three official tests authorized by the Japanese Orthopedic Association to evaluate the risk of *locomotive syndrome* (a condition of reduced mobility caused by an impairment of the locomotive organs). It has been reported that the Two-Step test score has a good correlation with one’s walking ability; however, its association with the gait pattern of older people during normal walking is still unknown. Therefore, this study aims to clarify the associations between the gait patterns of older people observed during normal walking and their Two-Step test scores.

**Methods:**

We analyzed the whole waveforms obtained from the lower-extremity joint angles and joint moments of 26 older people in various stages of locomotive syndrome using principal component analysis (PCA). The PCA was conducted using a 260 × 2424 input matrix constructed from the participants’ time-normalized pelvic and right-lower-limb-joint angles along three axes (ten trials of 26 participants, 101 time points, 4 angles, 3 axes, and 2 variable types per trial).

**Results:**

The Pearson product-moment correlation coefficient between the scores of the principal component vectors (PCVs) and the scores of the Two-Step test revealed that only one PCV (PCV 2) among the 61 obtained relevant PCVs is significantly related to the score of the Two-Step test.

**Conclusions:**

We therefore concluded that the joint angles and joint moments related to PCV 2—ankle plantar-flexion, ankle plantar-flexor moments during the late stance phase, ranges of motion and moments on the hip, knee, and ankle joints in the sagittal plane during the entire stance phase—are the motions associated with the Two-Step test.

**Electronic supplementary material:**

The online version of this article (10.1186/s12877-018-0784-5) contains supplementary material, which is available to authorized users.

## Background

The concept of *locomotive syndrome* was proposed by the Japanese Orthopedic Association (JOA); it typifies the condition of reduced mobility resulting from a locomotive organ disorder related to aging [1, 2]. The reduced mobility from musculoskeletal condition, such as knee arthritis, is considered as locomotive syndrome, while the reduced mobility from the neurological condition such as stroke, is not considered as locomotive syndrome. Further, the criteria for locomotive syndrome are the same for men and women. Degenerative changes in joints commonly leads to osteoarthritis of the knee and hip, as well as vertebral spondylosis. The dysfunction of the joint and related pain directly impair mobility, and the affected neuronal lesions adjacent to vertebral deformity also cause neurological symptoms. A recent clinical study reported that there are approximately 47 million people in Japan with radiographic knee osteoarthritis, lumber spondylosis, or osteoporosis, indicating they are suffering from, or will be suffering from the locomotive syndrome [3]. The Japanese Ministry of Health, Labor and Welfare reported that females have a higher risk of locomotive syndrome than males [4]. Walking is the most essential means of human mobility. Therefore, the detection of the symptoms related to locomotive syndrome and taking corrective measures in the early stages are important to lead and maintain an independent lifestyle.

The JOA prescribed three official tests for assessing the individual risk level of suffering from locomotive syndrome based on the evidence provided in previous studies [5–7] to detect the symptoms of locomotive syndrome in its early stage.: 1) the *stand-up* test to assess leg strength, 2) the *Two-Step test* to assess maximal stride length, and 3) a 25-question risk assessment questionnaire to assess the individual’s physical conditions and difficulties in daily life. The Two-Step test, which was first developed by Muranaga and Hirano in 2003 [7, 8], intended to assess one’s walking ability, including muscle strength, balance, and flexibility of the lower limbs [9]. Previous studies reported that the Two-Step test score was significantly associated with maximal gait speed [6], the risk of falling and degree of independence in daily life [10], gender (the scores of men were significantly higher than those of women) [9], and age (the scores were significantly lower for higher ages in both men and women) [9]. Despite the feasibility of the Two-Step test as a measure of mobility in locomotive syndrome, it is unknown which factors in gait pattern contribute to the score of the test. Revealing those factors, especially for relatively young older people, may provide novel insights for understanding the mechanisms underlying the declining mobility in the early stage of aging. In addition, the characterization of gait patterns of those who score low values in the Two-Step test will lead to the establishment of effective instruction to improve their gait performance in the early stage of locomotive syndrome.

Principal component analysis (PCA) is a technique that has recently attracted much interest in biomechanical studies, because of its usefulness in identifying the movement characteristics of various groups (and under various conditions) using whole data waveforms [11–18]. Nigg et al. [16] have recently pointed out that the success of the traditional gait assessment approach, which investigates a few selected variables at discrete time points, depends on the selection of variables made by the researchers, and can fail to detect potentially interesting results present in large portions of data left unanalyzed. Therefore, although there have been several studies to analyze the association between the Two-Step test score and gait parameters in older people [6, 9, 10], it is still unclear whether those analyses, in fact, revealed all the dominant components of gait features among the older people. PCA is a multivariate statistical technique that summarizes the information conveyed by many possibly correlated variables using a smaller number of uncorrelated variables (the principal components). PCA generates a set of principal component vectors (PCVs) and one set of principal component scores (PCSs) for each PCV. Each PCV corresponds to one of the orthogonal axes along which the variance of the data is maximal, and each PCS is the projection of the input data onto the corresponding PCV. In PCA, the movements with dominant differences (large variances) appear in the lower-numbered PCVs (and vice versa). Moreover, the data waveforms related to each PCV can be reconstructed (or *simulated*) by inputting the voluntary values as PCSs. Thus, if you input + 3 and − 3 standard deviation values as the PCS of certain PCV(s), exaggerated data waveforms related to the PCV(s) will be reconstructed, which make it easy to understand the gait features related to the PCV(s). Therefore, we conclude that PCA can be useful in capturing characteristics of gait patterns and here we attempt to clarify the associations between the gait patterns observed during normal walking and the scores of the Two-Step test.

A previous study [19] focused on the age-related step length reduction and determined that, compared to younger participants, older participants had smaller ankle plantar flexion, lower ankle plantar-flexor moments, and lower ankle plantar-flexor power during the late stance phase. In addition, the older people tended to compensate for these reductions by increasing the hip joint flexor moment and power. We expected a similar decline in the walking ability of older people with low scores on the Two-Step test, when compared to older people with high scores on this test. Therefore, we hypothesized that older people with low scores on the Two-Step test tended to exhibit smaller ankle plantar flexion and lower ankle plantar-flexor moments during the late stance phase, and that they would compensate for these reductions by increasing the hip joint flexor moment.

## Methods

### Participants

Walking gait data and Two-Step test scores were obtained from 26 older people in various stages of locomotive syndrome (9 males and 17 females), aged 60 to 74. In this study, we recruited relatively young older participants with more women than men, so that we can understand the associations between the gait patterns observed during normal walking and the scores of the Two-Step test in the early stage of aging population. The demographic data of the participants are presented in Table 1. All the participants were capable of walking independently without assistive devices (e.g., canes, crutches, or orthotic devices), had normal or corrected-to-normal vision, had no history of neuromuscular disease, and lived independently in their local communities. None of the participants had a history of surgery because of trauma or orthopedic diseases were excluded. Further, we excluded potential participants with any neurological disorder. The experimental protocol was approved by the local institutional review board (IRB), and all the participants gave their written informed consent before participating.

**Table 1:** Average (standard deviation) of the demographic data and spatio-temporal parameters of participants

Age [yrs]	Height [cm]	Body Mass [kg]	Step Length [cm]	Step Width [cm]
65.2 (3.5)	158.2 (8.3)	59.0 (10.4)	63.7 (6.3)	9.4 (3.1)
Stance Time [s]	Swing Time [s]	Cadence [step/min]	Walking Speed [m/s]	
0.58 (0.03)	0.40 (0.02)	61.65 (3.15)	1.32 (0.14)	

### Measurement

The Two-Step tests and the gait measurements were performed in a room with a straight 10-m path on which the participants could walk. Each participant was subjected to the Two-Step test prior to gait measurement.

During the Two-Step tests, all participants wore the same type of experimental wear (sleeveless shirt and spats) and shoes provided by the experimenter. The experimental wear and shoe sizes were selected by the participants themselves. Based on the instructions provided by JOA [20], each participant performed the Two-Step test twice, and the best score was recorded.

During gait measurement, the participants were asked to walk barefoot at a comfortable, self-selected speed. Three-dimensional (3D) positional data were obtained during the walk by using reflective markers and a 3D motion capture system (VICON MX, VICON, Oxford, UK) with a 200 Hz sampling frequency. A total of 57 infrared reflective markers were attached by one of three expert research assistants with more than 10 years of experience, in accordance with the guidelines of the Visual 3D software (C-Motion Inc., Rockville, MD, US). Simultaneously, ground reaction forces (GRFs) were obtained by using seven force plates (BP400600–2000, AMTI, Watertown, MA, US) sampled at 1 kHz. Before the walking trials, the positions of the markers were recorded while the participants stood stationary. The participants were then allowed sufficient practice walks to ensure a natural gait. After the practice, ten successful trials were recorded, in which each participant properly stepped on a force plate.

### Data analysis

The raw motion and GRF data were digitally filtered using a zero-lag, fourth-order, low-pass Butterworth filter; the filter cut-off frequencies were 10 Hz for the positional data and 56 Hz for the GRF data based on a previous study [21]. The angles of the hip, knee, and ankle joints, and the pelvis-link angle during one gait cycle were calculated for the x-axis (i.e., flexion–extension), y-axis (i.e., abduction–adduction), and z-axis (i.e., internal–external rotation) using a Cardan sequence of rotations (X-Y-Z) from the trajectories measured in each trial. The joint moments of the above-mentioned joints on the x-, y-, and z-axis during one gait cycle were calculated from the trajectories and GRFs measured in each trial, using Newton-Euler’s inverse-dynamics formula.

The angles and moments were time-normalized by the gait cycle duration determined from the force plate data and divided into 101 variables ranging from 0 to 100%. Therefore, each trial corresponded to a dataset of 2424 variables (101 time points, 4 angles in 3 axes, with 2 types of variables: moment and angle). The step length, step width, stance time, swing time, percentage of the stance-to-swing transition timing, cadence, and walking speed were also determined, to help understand the gait characteristics. The low-pass filtering and all variable calculations (i.e. joint and link angles, joint moments, and spatio-temporal parameters) were performed using the Visual 3D software package.

### Statistics

We applied PCA to the correlation matrix of the 2424 variables calculated from the 260 data points (ten trials for each of the 26 participants); the specific PCA procedure is described in the Appendix. The statistical analyses described below were conducted to identify the associations between the gait patterns observed during normal walking, as represented by the PCVs, and the scores of the Two-Step test. Additionally, for each PCV, simulated joint kinematic and kinetic waveforms were reconstructed from the PCSs with very large or very small values (deviating from the mean by three standard deviations), to interpret the joint angles and joint moments corresponding to the PCVs. These joint kinematic and kinetic waveforms were reconstructed using the technique presented by Kobayashi et al. [13, 14].

To determine the PCVs related to the Two-Step test scores, we calculated the Pearson product-moment correlation coefficient between the Two-Step test scores and the PCSs of the PCVs with contribution rates of 5% or more. Furthermore, to help understand the gait characteristics related to each PCV, we also calculated the Pearson product-moment correlation coefficient between the PCSs of the PCVs with contribution rates of 5% or more, the Two-Step test score, and seven different spatio-temporal parameters (step length, step width, stance time, swing time, percentage of the stance-to-swing transition timing, cadence, and walking speed). All statistical analyses were executed using the SPSS statistical software package (IBM SPSS Statistics Version 23, SPSS Inc., Chicago, IL, USA). Because of the large number of data points (*n* = 260), the correlation coefficients *r* were considered statistically significant if their values were greater than 0.3, indicating a medium effect size [22].

## Results

**Fig. 1:**
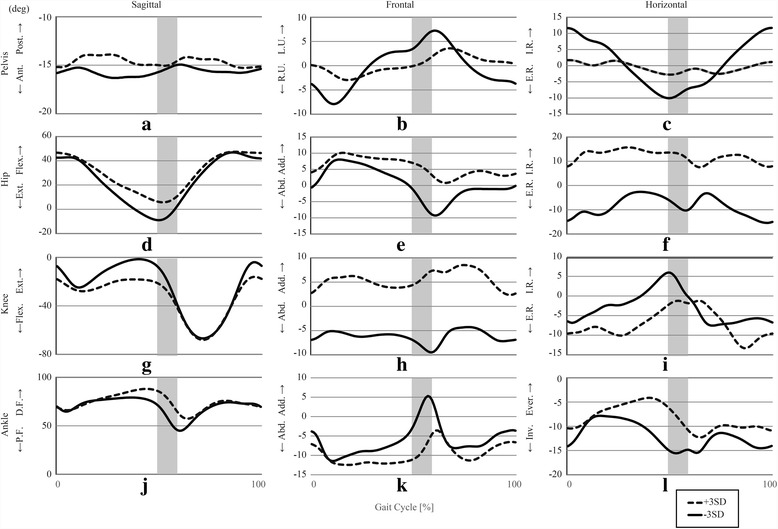
Joint kinematics recombined from the PCSs of PCV 2 The definitions of the abbreviations in the central tendency graph are as follows: Post.: Posterior. Tilt, Ant.: Anterior Tilt, Flex.: Flexion, Ext.: Extension, D.F.: Dorsi-flexion, P.F.: Plantar flexion, L.U.: Left Side Up, R.U.: Right Side Up, Add.: Adduction, Abd.: Abduction, I.R.: Internal Rotation, E.R.: External Rotation, Ever.: Eversion, Inv.: Inversion. Gray high-lighted area indicates the instance of the toe off (transition of stance phase and swing phase). It has certain width because present study did not separate stance phase from swing phase at the time-normalization procedure. Because there is a significant negative correlation between the PCVs and the Two-Step test scores, +3SD waveform can be interpreted as representing an exaggerated gait pattern consistent with older people with low scores on the Two-Step test, whereas -3SD waveform can be interpreted as representing an exaggerated gait pattern consistent with older people with high scores on the Two-Step test. **a** sattigal pelvis angle, **b** frontal pelvis angle, **c** horizontal pelvis angle, **d** sagittal hip angle, **e** frontal hip angle, **f** horizontal hip angle, **g** sagittal knee angle, **h** frontal knee angle, **i** horizontal knee angle, **j** sagittal ankle angle, **k** frontal ankle angle, **l** horizontal ankle angle

**Fig. 2:**
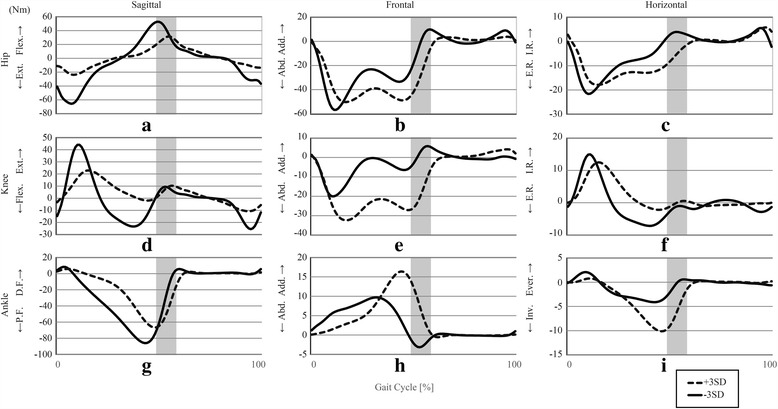
Joint kinetics recombined from the PCSs of PCV 2 The definitions of the abbreviations in the central tendency graph are as follows: Post.: Posterior. Tilt, Ant.: Anterior Tilt, Flex.: Flexion, Ext.: Extension, D.F.: Dorsi-flexion, P.F.: Plantar flexion, L.U.: Left Side Up, R.U.: Right Side Up, Add.: Adduction, Abd.: Abduction, I.R.: Internal Rotation, E.R.: External Rotation, Ever.: Eversion, Inv.: Inversion. Gray high-lighted area indicates the instance of the toe off (transition of stance phase and swing phase). It has certain width because present study did not separate stance phase from swing phase at the time-normalization procedure. Because there is a significant negative correlation between the PCVs and the Two-Step test scores, +3SD waveform can be interpreted as representing an exaggerated gait pattern consistent with older people with low scores on the Two-Step test, whereas -3SD waveform can be interpreted as representing an exaggerated gait pattern consistent with older people with high scores on the Two-Step test. **a** sagittal hip moment, **b** frontal hip moment, **c** horizontal hip moment, **d** sagittal knee moment, **e** frontal knee moment, **f** horizontal knee moment, **g** sagittal ankle moment, **h** frontal ankle moment, **i** horizontal ankle moment

The PCA revealed that the first 61 PCVs explained more than 98% of the total variance. This study focused on the first 19 of these PCVs, each of which explained more than 1% of the total variance of the 260 gait samples. Together, these first 19 PCVs explained 87.161% of the variance. The explained variance and the Pearson product-moment correlation coefficients between the PCSs of the first 19 PCVs are shown in Table 2, along with the Two-Step test scores for each PCV. As shown, a significant correlation between the PCVs and the Two-Step test scores was found only on PCV 2 (− 0.445). Figures 1 and 2 show the reconstructed joint kinematic and kinetic waveforms of PCV 2 by presenting the pelvic, hip-joint, knee-joint, and ankle-joint angles and moments along the sagittal, frontal, and horizontal planes; very large (three standard deviation interval) positive (dotted lines) and negative (solid lines) deviations are also illustrated. For the PCV 2, older people with low scores on the Two-Step test tended to exhibit larger PCSs than older people with high scores on the Two-Step test. Therefore, the reconstructed waveforms indicated by the dotted lines in Figs. 1 and 2 (corresponding to very large positive deviations), can be interpreted as representing an exaggerated gait pattern consistent with older people with low scores on the Two-Step test. Similarly, the reconstructed waveforms indicated by the solid lines in Figs. 1 and 2 (corresponding to very large negative deviations), can be interpreted as representing an exaggerated gait pattern consistent with older people with high scores on the Two-Step test. We also provide a stick figure animation of the lower limb movements showing the gaits for PCV 2, to help understand how this PCV affects the joint angles and joint moments (see the Additional file 1).

**Table 2:** Results of main PCA. The main PCA generated a total of 19 PCVs to achieve a cumulate description of 87.161% of the variability, and only PCV 2 revealed significant correlation with the scores on the two-step test

	PCV1	PCV2	PCV3	PCV4	PCV5	PCV6	PCV7	PCV8	PCV9	PCV10
Explained variance (%)	18.895	9.696	9.379	7.844	5.747	4.946	4.494	3.948	3.754	2.985
Cumulative (%)	18.895	28.591	37.970	45.814	51.561	56.508	61.002	64.950	68.704	71.689
*r*	−0.250	−0.445	0.010	0.252	0.083	0.149	−0.033	0.134	−0.053	− 0.116
	PCV11	PCV12	PCV13	PCV14	PCV15	PCV16	PCV17	PCV18	PCV19	
Explained variance (%)	2.550	2.378	2.132	1.786	1.753	1.385	1.280	1.185	1.025	
Cumulative (%)	74.238	76.616	78.748	80.534	82.287	83.672	84.952	86.136	87.161	
*r*	0.007	0.144	0.148	−0.171	−0.041	0.272	0.077	−0.002	−0.017

The Pearson product-moment correlation coefficient revealed that the PCSs of PCV 2 were significantly correlated with the step length (*r* = − 0.686), swing time (*r* = − 0.375), percentage of the stance-to-swing transition timing (*r* = − 0.318), and walking speed (*r* = − 0.553) (Table 3). Further, the scores of the Two-Step test were significantly correlated with the step length (*r* = 0.319), cadence (*r* = 0.323), and walking speed (*r* = 0.319) (Table 3).

**Table 3:** Pearson’s product moment correlation between the PCSs of PCV 2 and spatio-temporal parameters

*r*	Step Length	Step Width	Stance Time	Swing Time	% of the stance-to-swing transition timing	Cadence	Walking Speed
*PCV 2*	−0.686	−0.204	0.033	−0.375	− 0.318	0.139	− 0.553
*Two-Step test score*	0.319	−0.049	−0.067	0.234	−0.043	0.323	0.319

## Discussion

The objective of this study was to use PCA to clarify the associations between the gait pattern during normal walking and the Two-Step test scores. We initially hypothesized that older people with low scores on the Two-Step test tended to exhibit smaller ankle plantar-flexion and lower ankle plantar-flexor moments during the late stance phase, and that they would compensate for these reductions by increasing the hip joint flexor moment. The results of the present study confirm this hypothesis partially, as described below.

A significant negative correlation between the PCSs and the Two-Step test scores was observed only on PCV 2. In PCA, the movements with dominant differences appear in the lower-numbered PCVs. Therefore, it is reasonable to interpret the motions related to PCV 2—which are shown in Figs. 1 and 2—as the gait pattern characteristics associated with the Two-Step test score.

As expected, the reconstructed waveforms (see Figs. 1 and 2) revealed a trend in which older people with low scores on the Two-Step test exhibited smaller ankle plantar-flexion and lower ankle plantar-flexor moments during the late stance phase than older people with high scores on the Two-Step test. These results are similar to the ones of a previous study [19] comparing joint angles and joint moments between healthy young people and older people. However, they did not compensate for these reductions by increasing the hip joint flexor moment. Furthermore, the reconstructed waveforms reveal that older people with low scores on the Two-Step test tend to exhibit a smaller range of motions and moments on the hip, knee, and ankle joints in the sagittal plane during the entire stance phase, when compared to older people with high scores on the Two-Step test. These results indicate that older people with low scores on the Two-Step test have difficulty maintaining hip motion in the sagittal plane, which is the well-recognized compensation for weak plantar flexion [19], and the Two-Step test can detect such difficulty from the early stage of aging. We assume that this failure in compensating the weak plantar flexion leads to a further reduced ability to progress the body forward during normal walking.

A significant negative correlation was found between the PCSs of PCV 2 and both the swing time (*r* = − 0.375) and the percentage of the stance-to-swing transition timing (*r* = − 0.318). These results indicate that older people with large PCSs on this PCV (i.e., older people with low scores on the Two-Step test) tend to exhibit shorter swing times and smaller percentages of swing phase in one gait cycle than older people with small PCSs on this PCV (i.e., older people with high scores on the Two-Step test). Indeed, some of the figures clearly show a phase shift between the waveforms during the late stance phase. For example, in Fig. 1j, the local minima of the dotted line appear later than those of the solid line.

Most of the traditional gait studies investigated the selected variables only at discrete time points; large portions of the data were therefore not analyzed. PCA, however, can both analyze the whole waveforms and emphasize the gait characteristics of the target populations. These advantages of using PCA enable us to understand and discuss a very comprehensive summary of the gait characteristics associated with the Two-Step test scores. However, there are some factors that must be considered when interpreting the results of the current study. First, we must note that soft tissue artifacts may cause a bias in the observed plane angles, especially in the hip and knee joints. Although we placed markers on the body’s bony landmarks, such artifacts must exist. Second, the judgments concerning the significance of correlations were made based on whether the *r*-values were greater than 0.3, because of the large number of data points (*n* = 260). These limitations may affect the generality of the results of the present study. Therefore, further studies to evaluate and overcome these limitations may still be required. Further, it is noteworthy that participants in this study were relatively young (mean age 65.2 years) and the gait speeds were quite fast (1.32 m/s on average). Therefore, as for clinical implications, the current study shows that the Two-Step test, a newly proposed test for evaluating the presence of the locomotive syndrome, reflects the specific gait pattern and the walking speed even for the relatively young older people. Further accumulation of data and PCA analyses should provide new insights concerning the trajectory of gait deterioration among older people, especially in the case of those with locomotive organ diseases.

## Conclusions

This study used PCA to clarify the associations between the gait patterns during normal walking and the Two-Step test scores. The whole waveforms obtained from the lower-extremity joints’ kinematics and kinetics of 26 older people in various stages of the locomotive syndrome were analyzed using PCA. The Pearson product-moment correlation coefficient between the PCSs and the scores of the Two-Step test revealed that only one PCV (PCV 2) among the 61 obtained relevant PCVs was significantly related to the Two-Step test scores. Therefore, we concluded that the joint angles and joint moments related to PCV 2—ankle plantar-flexion, ankle plantar-flexor moments during the late stance phase, range of motions and moments on the hip, knee, and ankle joints in the sagittal plane during the entire stance phase—are the motions associated with the Two-Step test.

### Additional file

Additional file 1: Stick figure animation of the lower limb movements relates to PCV 2. This file provides a stick figure animation of the lower limb movements showing the gaits for PCV 2. (GIF 1043 kb)

## Appendix

### PCA procedure adopted in this study.

The PCA analysis conducted as part of this study proceeded as follows. First, we calculated the centered mean of each of the 2424 variables for centering and scaling by using following formula:

1 $$ {z}_{\mathrm{t}\mathrm{n}}=\left({\mathrm{X}}_{\mathrm{t}\mathrm{n}}-{\mu}_{\mathrm{t}}\right)/{\sigma}_{\mathrm{t}} $$

where z_tn_ is the scaled data for parameter t and trial n, X_tn_ is the raw data for parameter t and trial n, μ_t_ is the mean of parameter t across all trials, and σ_t_ is the standard deviation of that same parameter across all trials. The parameter index t ranges between 1 and 2424 and the parameter index n ranges between 1 and 260. Second, we constructed a 260 × 2424 input matrix containing the ten trials of each of the 26 participants, and their respective 2424 mean-centered parameters. Third, we performed a PCA on the input matrix by using a correlation matrix. Fourth, we conducted the statistical analysis described in the *Statistics* section above, to identify the PCVs related to the scores of the Two-Step test. Finally, the joint angle and moment waveforms were reconstructed by applying the technique proposed in [9] to interpret the corresponding gait characteristics related to the scores of the Two-Step test.

## Abbreviations

GRF: Ground reaction force
JOA: Japanese Orthopaedic Association
PCA: Principal component analysis
PCV: Principal component vector
PSC: Principal component score
