# Supplementary figures and images for: Association between the gait pattern characteristics of older people and their two-step test scores
Source: BMC Geriatr. 2018 Apr 27;18:101. doi: 10.1186/s12877-018-0784-5 (PMC5921743; doi:10.1186/s12877-018-0784-5)

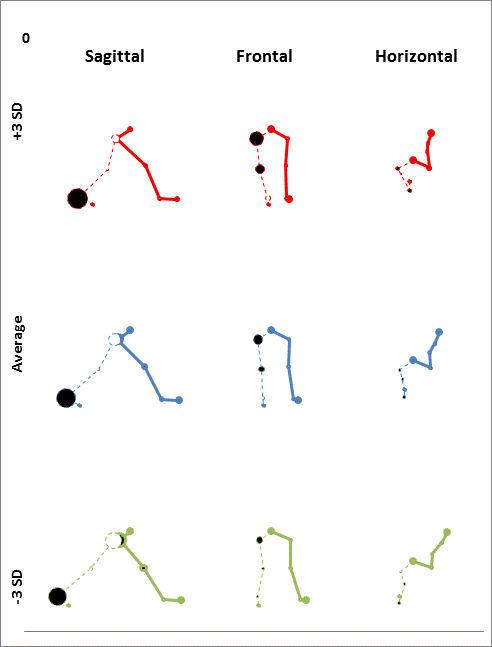

Supplement: Supplementary file 1 — Stick figure animation of the lower limb movements relates to PCV 2. This file provides a stick figure animation of the lower limb movements showing the gaits for PCV 2. (GIF 1043 kb) [file 12877_2018_784_MOESM1_ESM.gif]
